# RNA sequencing data of tobacco inoculated with *Apple stem pitting virus*

**DOI:** 10.1016/j.dib.2019.105106

**Published:** 2020-01-03

**Authors:** Gengxuan Yan, Hongyi Yang

**Affiliations:** a Key Laboratory of Saline-alkali Vegetation Ecology Restoration (Northeast Forestry University), Ministry of Education, Harbin 150040, China; b College of Life Sciences, Northeast Forestry University, Harbin 150040, China

**Keywords:** Tobacco, Next-generation sequencing, RNA-seq, *Apple stem pitting virus*

## Abstract

*Apple stem pitting virus* (ASPV) mainly infects apple, pear and their closely related species in the world. ASPV causes some symptoms like leaf etiolation and stony pit in cultivated pear, but produces few symptoms in cultivated apple. We inoculated tobacco with ASPV, which originates from Nanking cherry (*Prunus tomentosa*), and we obtained tobacco RNA-sequencing data through high-throughput sequencing. In total, 17,401,736 clean reads were obtained after sequencing and removing adaptor sequences, contamination and low-quality reads. An RNA-seq data has been uploaded to Sequence Read Archive (SRA), which enables researchers to access the RNA-sequencing data of tobacco inoculated with ASPV.

**Table:** Specifications Table

Subject	Plant Science
Specific subject area	Plant disease
Type of data	RNA Sequencing Data
How data were acquired	The data acquired by Next-generation Sequencing technology using Illumina HiSeq 4000 platform.
Data format	Raw sequences (FASTQ)
Parameters for data collection	Total RNA was obtained from tobacco leaves inoculated with ASPV.
Description of data collection	The RNA was sequenced with Next Generation Sequencing method using Illumina HiSeq 4000 platform in BGI tech, Shenzhen.
Data source location	Harbin, Heilongjiang Province, China (N 45° 43′, E 126° 37′)
Data accessibility	Raw data of RNA Seq analysis are available on Sequence Read Archive (SRA) database and connected to BioProject PRJNA579004, https://www.ncbi.nlm.nih.gov/bioproject/PRJNA579004.

**Table:** 

**Value of the Data** • ASPV is one of the latent viruses causing apple stem pox disease, pear vein yellow disease, pear stone pox disease and other diseases with less effective control approach. • This data provides the RNA sequencing data of tobacco leaves inoculated with *Apple stem pitting virus.* • This data contains sequence information of *Apple stem pitting virus.* It is advantageous to analyse the variation of *Apple stem pitting virus.*

## 1 Data description

ASPV is known to infect apple, pear and their closely related species like quince, hawthorn and mountain ash [1]. Some new ornamental plant host of ASPV like *Cydonia japonica*, *Pyrus calleryana* and *Pyrus amygdaliformis* were reported in recent few years [2]. ASPV can be spread to *Nicotiana occidentalis* and its subspecies through friction inoculation [3]. ASPV can cause various symptoms in different host plants, such as leaf etiolation in most species of pear and fruit shrink in some cultivars of apple [4].

Genome of ASPV is a single-stranded linear RNA molecules of 12–15 nm × 800 nm in size [5]. The typical isolate of ASPV, PA66 genome consists of 9306 nucleotides including five open reading frame (ORFs). In this five ORFs, ORF1 encodes about 247KD replicase protein associated with virus replication, ORF2 to ORF4 constitute gene expression cassettes respectively encoding 25KD, 13KD, 7KD triple gene block protein which promotes virus transmission in host plants collectively, while ORF5 encodes 44KD coat protein to wrap virus nucleic acids and participate in host recognition [6]. It is reported that there is a higher degree of genetic variability in coat protein among different ASPV isolates [7]. Therefore, analysis of coat protein gene mutation shows a way to research phylogenetic process of ASPV in diverse hosts.

The variation of ASPV is very complicated. In addition, it is difficult to isolate high quality nucleic acid from the tissue of Nanking cherry (*Cerasus tomentosa*), which contains plenty of secondary metabolites, such as polyphenols and polysaccharides. We have reported ASPV infected Nanking cherry, however, we cannot amplify more genome region by RT-PCR from Nanking cherry [8]. Therefore, we transferred ASPV from Nanking cherry to tobacco by friction inoculation. Finally, we try to obtain more sequence information of the ASPV isolate of Nanking cherry from tobacco RNA-sequencing data through high-throughput sequencing.

**Fig. 1:**
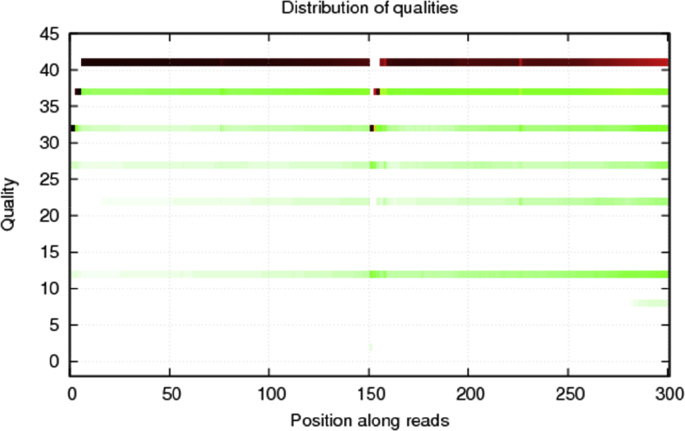
Distribution of qualities along reads after filtering of the sample.

After sequencing, the raw reads were filtered. Data filtering includes removing adaptor sequences, contamination and low-quality reads from raw reads. Next, we get the statistics of data production. A total of 17,401,736 clean reads of 150 bp length were obtained from re-sequencing project, while in these clean data, percentage of the number of nucleotides with quality higher than 20- nucleotide (Q20) is 96.26%, and GC content among all four kinds of bases is 39.35%. The quality control of sample shows in Fig. 1.

## 2 Experimental design, materials, and methods

### 2.1 Inoculation

Nanking cherry sample was obtained from Heilongjiang province of China in 2013, and ASPV was detected by RT-PCR and ELISA [8]. ASPV was transferred from Nanking cherry to tobacco through friction inoculation. Two gram of ASPV infected leaves of Nanking cherry were collected to prepare inoculation. Leaves were fully grinded with 10ml phosphate buffered saline (PBS) before filtered to keep the filtrate [9]. When tobaccos grew to 4–5 leaves, a little bit of filtrate were dripped to leaves, and sprinkled some silicon carbide on the surface of tobacco leaf. The leaves were rubbed by finger. Finally, wash the leaf by sterile water until silicon carbid on the leaf surface is fully washed away. Tobacco was grown for 25 °C under 16h of light (700 lux) each day. To acquire the presence of ASPV traceability, RT-PCR was used to confirm the presence of ASPV in tobacco leaves after 3-week inoculation. ASPV was detected by RT-PCR using a pair of primers A/C (5′-ATAGCCGCCCCGGTTAGGTT-3′; 5′-CTCTTGAACCAGCTGATGGC-3′) [10]. Virus-specific origin of the amplicons was confirmed by cloning and sequencing. Additional RT-PCR using a sense primer SPcp1 (5′-AGYGAGCCAGTSATHTCTCA-3′) [11]and an M4 primer (5′-GTTTTCCCAGTCACGAC-3′) [12] was conducted to amplify the partial coat protein (CP) gene and 3′ untranslated region of the virus. Collect the inoculated tobacco leaves and store them in liquid nitrogen.

### 2.2 RNA extraction

Frozen tissue was grinded into fine powder in liquid nitrogen before RNA extraction. The total RNA was extracted from 100mg tissue sample using the RNAprep pure Plant Kit (TIANGEN, Beijing, China) referred to manufacturer's instruction. RNA from plant tissue was fixed in the spin column after extraction by chloroform. Then, several washing steps were proceeded to elute genome RNA from spin column. RNA was concentrated by ethanol and stored in TE buffer. The RNA was analysed by agarose gel electrophoresis, while the concentration of RNA was determined using the Qubit Fluorometer [13] (Invitrogen, Carlsbad, CA, USA).

### 2.3 Library construction and sequencing

Total RNA was extracted and treated with DNase I. Then cDNA is synthesized using the mRNA fragments as templates. Short fragments are purified and resolved with EB buffer for end reparation and single nucleotide A (adenine) addition. After that, the short fragments are connected with adapters. The suitable fragments are selected for the PCR amplification. Then, A 800bp insert library was constructed and sequenced on an Illumina HiSeq 4000 platform [14]. To obtain high quality reads from high-throughput sequencing, we filtered out the low-quality reads depending on the following criteria: (a) reads with >2% unidentified nucleotides (N) or with poly-A structure; (b) reads with over than 40% bases having low quality for short insert-size libraries and more than 60% for large insert-size libraries; (c) reads with adapters or PCR duplication; (d) reads with 20 bp in 5′ terminal and 5 bp in 3’ terminal [15]. In total, 17,401,736 clean reads were obtained for local alignment.
